# Delving into Agri-Food Waste Composition for Antibacterial Phytochemicals

**DOI:** 10.3390/metabo13050634

**Published:** 2023-05-07

**Authors:** Jorge A. M. Pereira, Cristina V. Berenguer, José S. Câmara

**Affiliations:** 1 CQM—Centro de Química da Madeira, Campus da Penteada, Universidade da Madeira, 9020-105 Funchal, Portugal; 2 Departamento de Química, Faculdade de Ciências Exatas e da Engenharia, Campus da Penteada, Universidade da Madeira, 9020-105 Funchal, Portugal

**Keywords:** agri-food wastes, phytochemicals, multidrug resistance, bacterial infection, antibacterial activity

## Abstract

The overuse of antibiotics in the healthcare, veterinary, and agricultural industries has led to the development of antimicrobial resistance (AMR), resulting in significant economic losses worldwide and a growing healthcare problem that urgently needs to be solved. Plants produce a variety of secondary metabolites, making them an area of interest in the search for new phytochemicals to cope with AMR. A great part of agri-food waste is of plant origin, constituting a promising source of valuable compounds with different bioactivities, including those against antimicrobial resistance. Many types of phytochemicals, such as carotenoids, tocopherols, glucosinolates, and phenolic compounds, are widely present in plant by-products, such as citrus peels, tomato waste, and wine pomace. Unveiling these and other bioactive compounds is therefore very relevant and could be an important and sustainable form of agri-food waste valorisation, adding profit for local economies and mitigating the negative impact of these wastes’ decomposition on the environment. This review will focus on the potential of agri-food waste from a plant origin as a source of phytochemicals with antibacterial activity for global health benefits against AMR.

## 1. Introduction

Food waste is an inevitable outcome of food production processes, and substantial quantities of by-products are generated along the food chain. According to the United Nations Food and Agriculture Organization (FAO), approximately one-third of all food produced for human consumption globally is wasted at various stages of the food supply chain and during production, post-harvest handling and storage, processing, distribution, and consumption [1]. 

Such waste, estimated at 1.3 billion tonnes of food per year [1], constitutes a burden for the industry, which often has to pay to discard it. Moreover, there are obvious economic and environmental impacts, particularly when the wastes are deposited in landfills with no or minimal processing, contributing to greenhouse gas emissions and groundwater contamination [1]. This scenario will gradually worsen as the human population continues to grow exponentially, and, despite the huge amounts of wasted food, more food will be produced.

In general terms, agri-food waste refers to any organic material that is discarded during food production or processing activities. This can include everything from animal and plant by-products, such as bones and fat, to leftover crops or spoiled produce that cannot be sold for human consumption. Most of this food wastage, particularly that of a plant origin, contains considerable amounts of phytochemicals with interesting bioactivities for animal and human health management. This includes valuable compounds with the potential to generate enough revenues for the valorisation of agri-food residues. This is an important goal that can greatly benefit the environment and improve the food chain’s security and sustainability [2]. This strategy requires a deeper understanding of the composition of agri-food waste and the properties of its main components to unveil potential uses and routes for waste processing, envisaging a zero-waste policy promoted, for instance, by EU authorities [3]. As an example of such a strategy, it is worthwhile to refer to the work of Šeregelj et al. [4]. The authors encapsulated red pepper waste bioactive compounds, which were used to develop a functional yoghurt without losing its original sensorial properties. D-phytochemicals were found in red pepper wastes, namely carotenoids (β-carotene, lutein, zeaxanthin, β-cryptoxanthin), hydroxybenzoic acids (gallic, vanillic, protocatechuic acid), hydroxycinnamic acids (sinapic, caffeic, rosmarinic, chlorogenic acid), flavan-3-ols (epicatechin), and flavonols (rutin, quercetin, and myricetin). The fortification of the yoghourt had a positive influence on maintaining the initial number of lactic acid bacteria during storage, which retained carotenoids and increased polyphenol retention.

Antimicrobial resistance (AMR) involving the transfer of bacteria and genes between humans, animals, and the environment constitutes a global challenge [5], and researchers are actively trying to unveil new drugs able to mitigate this health problem. As referred to above, plants produce a variety of secondary metabolites, and a substantial part of agri-food waste is from a plant origin. Therefore, there is great potential in delving into agri-food waste composition to unveil potential antibacterial phytochemicals. In this context, this review will briefly refer to the impact of bacterial activity in the food chain and human health and the challenges posed by AMR, discussing then the use of extraction and chromatographic technologies to identify and quantify antibacterial phytochemicals in agri-food wastes and how these phytochemicals can be used as alternative antibiotics and food additives. To obtain a focused discussion of this topic, only applications reported in the literature since 2018 were considered.

## 2. The Impact of Bacterial Activity

### 2.1. Most Relevant Bacteria Affecting Human and Animal Health, Food Preservation, and Environment

**Figure 1:**
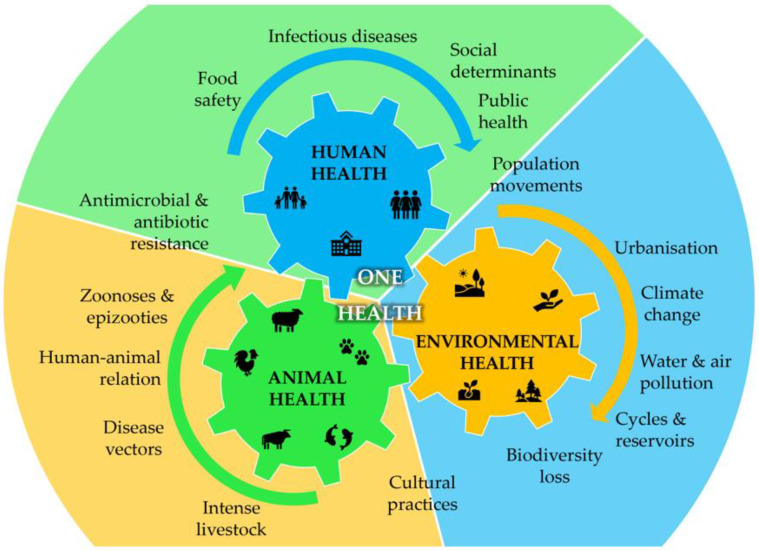
Integrated overview of the One Health concept showing the interdependence between human, animal, and environmental health and the interplay of the main factors that drive each one of the three categories (adapted from [7,8,9]).

Bacteria are present everywhere, including in humans, where there are roughly as many bacteria as host cells [6]. Most of these bacteria are essential to host metabolism, and a delicate balance is established between host and foreign cells to maintain homeostasis. However, human activity in the environment continually puts pressure on the human-animal–ecosystems interface, leading to the disruption and extinction of natural ecosystems and species that have evolved over millions of years. Recently, this has been acknowledged through the One Health initiative, which aims to oversee the challenges for human health integrated holistically with animal and environmental health [7,8,9]. Zoonotic diseases caused by bacterial outbreaks, for instance, are easily transmitted through contact with animals, food, water, and contaminated environments, emerging as serious challenges to public health. These zoonoses can easily affect aquaculture, agriculture, and other food systems [10], disrupting the food chain supply to millions of people on the planet (Figure 1). Several bacteria are more prone to elicit the referred problems, mostly by causing food poisoning and environmental contamination or directly infecting the human host. Table 1 describes the most important bacteria causing food poisoning and environmental contamination or acting directly as human pathogens. Food spoilage from bacteria is often accompanied by a decay in the sensory attributes that makes consumers reject ingestion. However, cross-contamination and toxin production by pathogenic microorganisms present in food is not so easily observed and poses risks to consumers. Raw or poorly processed foods, such as milk and dairy products, meat, and poultry, can be contaminated with different bacteria, such as *Bacillus cereus* [11], *Brucella* spp. [12], *Campylobacter* spp. [13], *Clostridium difficile* (*C. difficile*) [14], *Escherichia coli* (*E. coli*) [15], *Staphylococcus aureus* (*S. aureus*) [16], or *Yersinia* spp. [17,18]. In turn, eggs, poultry, and meat are more often contaminated with *Salmonella* spp. [19,20] and seafood with *C. difficile* [14], *Listeria monocytogenes* [21,22], or *Vibrio* spp. [23,24,25]. These infections may cause different alterations in the host depending on the susceptibility and severity of the pathogen contamination, ranging from asymptomatic contaminations to transitory digestive alterations (gastroenteritis, diarrhoea, vomiting, nausea, and mild fevers), or the infections can even affect other systems (hepatobiliary, genitourinary, musculoskeletal, cardiovascular, and integumentary systems), representing a serious threat to humans. These harsh effects are often associated with toxigenic strains such as *C. difficile*, *E. coli*, *S. aureus*, or *V. cholerae* (Table 1), whose toxins can be very harmful to the infected host. There are, however, other bacteria that form spores to withstand unfavourable conditions, such as *B. cereus* [11]. In this context, the combination of these two characteristics, which occurs with *C. difficile*, can make this bacterial infection particularly dangerous. *C. difficile* can survive in harsh conditions (e.g., antibiotic therapy) and later, under favourable conditions, produce toxins soon after the germination of the spores, causing serious illness or even death, especially in vulnerable populations, such as elderly people and those with weakened immune systems. The effect of food poisoning caused by bacteria on human health is, therefore, broad, and it is even more dangerous when the infection is caused by bacteria that evolved as human pathogens. As shown in Table 1, several bacteria can cause severe disruptions in human health. Some of them, such as *Acinetobacter baumannii*, *Klebsiella pneumoniae*, or *Pseudomonas aeruginosa* (*P. aeruginosa*), are opportunistic bacteria populating clinical environments and infecting immunocompromised patients [26,27,28]. Others, such as the *Neisseria* strains *Neisseria meningitidis* and *Neisseria gonorrhoeae*, can cause serious infections in humans, such as meningitis or gonorrhoea, respectively. Finally, *Mycobacterium tuberculosis* is responsible for tuberculosis (TB), a primary respiratory and incapacitating infection. TB is the leading cause of death caused by a single infectious agent [29].

### 2.2. The Challenge of Antimicrobial Resistance (AMR)

AMR is a significant problem in the field of medicine and healthcare. It refers to microorganisms, mainly bacteria, viruses, and fungi, that become resistant to multiple drugs and treatments that were primarily designed to target them. This is often the result of the indiscriminate use of antibiotics in the healthcare, veterinary, and agricultural industries [68,69]. Inadequate antibiotic and dosing choices and unnecessarily extended treatment have also contributed to the problem. Overall, these strategies have boosted antibiotic resistance in different environments, such as hospitals, nursing homes, and communities [70]. Consequently, AMR has become a major public health challenge, described by the World Health Organization as one of the top 10 public health challenges worldwide. In 2019 alone, it was estimated that 4.95 million deaths were associated with bacterial antimicrobial resistance, with 1.27 million being a direct cause of bacterial antimicrobial resistance [71]. These figures are concerning because the treatment of infectious diseases involving AMR will become progressively harder with a limited portfolio of effective drugs. As a result, prohibitive healthcare costs, morbidity, and mortality rates will likely increase soon [72]. Antibiotic resistance is not only a serious threat to humans but also to the environment. The use of antibiotics in food-producing animals is already a major public health problem that needs to be addressed. Different antibiotics have been used not only to prevent and treat infectious diseases but also to promote faster growth and higher productivity, further inducing and spreading antibiotic resistance between animals and from animals to humans [69]. Ultimately, this can result in environmental antibiotic resistance that can affect human health because the consumption of food contaminated with both pathogenic and nonpathogenic bacteria facilitate the transfer of antibiotic resistance among bacterial strains [69,73]. Bacteria can adapt to antibiotic resistance through two major genetic strategies, mutational resistance and horizontal gene transfer [5,74]. In mutational resistance, a subset of bacterial cells derived from a susceptible population develop mutations in gene(s) associated with the mechanism of action of the compound or drug [74]. Consequently, the resistant mutant will show preserved cell survival in the presence of the antimicrobial molecule, resulting in antimicrobial resistance. Acquired mutational changes are diverse and vary in complexity and include modification of the antimicrobial target, leading to a decreased affinity for the drug [74] and, thus, a decreased drug uptake: activation of efflux mechanisms to expel the antimicrobial molecule [75], loss of porin proteins preventing the accumulation of antimicrobial drugs [76], and important changes in metabolic pathways via the modulation of regulatory networks [5,77,78,79]. The acquisition of foreign DNA material for resistance determinants through horizontal gene transfer is also responsible for antimicrobial resistance. Horizontal gene transfer can occur through different strategies, namely transformation, transduction, or conjugation. Integrons are another important mechanism for accumulating antimicrobial resistance genes, representing one of the main drivers of bacterial evolution [5,79,80]. Moreover, as some of these bacteria are essential to our metabolism, there are growing concerns that human microbiota can also be severely affected by resistant bacteria, further increasing the burden of AMR in the health systems [81]. Currently, the most common multidrug-resistant bacteria include methicillin-resistant *Staphylococcus aureus* (MRSA) [82], vancomycin-resistant *Enterococcus* (VRE) [83], extended-spectrum beta-lactamase-producing *Enterobacteriaceae* (ESBL) [84], carbapenem-resistant *Enterobacteriaceae* (CRE) [85], *P. aeruginosa* [86], *Acinetobacter baumannii* [87], *Klebsiella pneumoniae* [88], *Mycobacterium tuberculosis* [55], *Salmonella enterica* [89], and *Neisseria gonorrhoeae* [62]. These bacteria develop different strategies to overcome the host’s defences, which can occur mainly in three types: efflux-mediated multidrug resistance, target modification, or enzymatic inactivation [72,90]. The efflux-mediated multidrug resistance involves the expulsion of drugs from the cell by pumps that are normally responsible for removing toxic molecules and waste products. Another type of AMR is target modification [72]. In this type of resistance, the target of the drug is modified so that it can no longer recognize or bind to the drug, rendering it ineffective. This type of resistance is commonly observed in bacteria and viruses where they alter the structure or expression of the target molecule to evade drug action. Finally, enzymatic inactivation occurs when microorganisms produce enzymes that can break down or modify the drugs before they can reach their intended target. This mechanism is particularly common in bacteria, which may produce enzymes such as β-lactamases that can degrade antibiotics like penicillin and cephalosporins [72]. Overall, multidrug resistance is a complex phenomenon that poses significant challenges to the development of effective treatments for infectious diseases. Efforts are underway to tackle this problem through the development of new drugs and alternative treatment strategies, such as combination therapy and immunotherapy [91,92,93]. 

## 3. Antibacterial Phytochemicals Identified in Food Wastes

Phytochemicals are natural chemical compounds found in plant foods, such as fruits, vegetables, legumes, whole grains, nuts, seeds, and herbs. These compounds act as a natural defence system for plants, protecting them from infections and microbial invasions and giving them colour, aroma, and flavour [94]. Phytochemicals have emerged as safe alternatives to conventional antibiotics to treat antibiotic-resistant pathogen-originated infections, as well as an alternative to chemical additives to foodborne bacteria [95]. Many phytochemicals have demonstrated their potential as bactericidal agents and have proved to inhibit the vital events for the sustenance and resistance of the pathogen, including efflux pumps, replication machinery, and cell permeability, among others [96]. Phytochemicals are grouped according to their structural characteristics into four large groups: nitrogen alkaloids, phenolic compounds, terpenoids, and organosulfur compounds [94]. 

**Figure 2:**
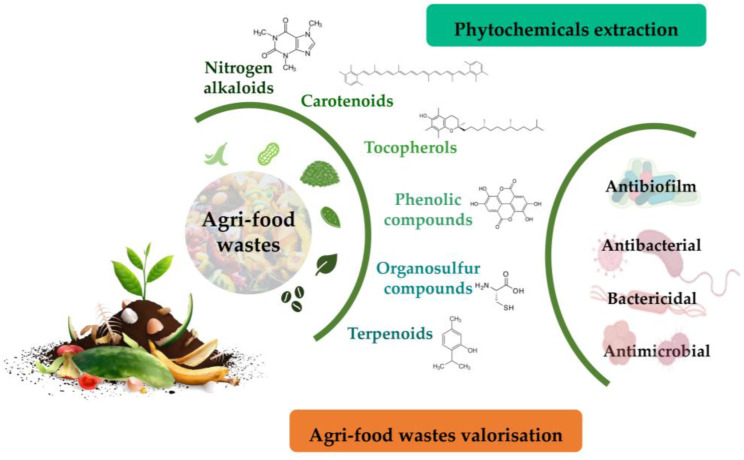
Overall strategy to unveil phytochemicals with antimicrobial activity from food wastes as a strategy for their valorisation.

Agri-food wastes comprise peels, seeds, shells, pomace, and leaves. These residues are important substrates for phytochemicals, including polyphenols, carotenoids, essential oils, tocopherols, and terpenes. In addition to their antibiotic activities, phytochemicals found in agri-food wastes can be easily managed via their valorisation to produce value-added products, food additives, therapeutics, or other environmental applications due to their antioxidant, therapeutic, and nutritional properties [97,98,99,100] (Figure 2). 

Several studies have shown the antibacterial potential of phytochemicals found in agri-food wastes (Table 2). For instance, Carmo and collaborators [101] isolated coumarins (bergapten, xanthotoxin, dimethyl allyl xanthyletin) and an imidazole alkaloid from the crude extract of leaves and bark of *Pilocarpus pennatifolius* Lemaire. The extracts and pure compounds were tested against different strains of bacteria and fungi, which showed promising antimicrobial and antifungal activities. The alkaloid identified showed a minimal inhibitory concentration of 1.56 μg·mL^−1^ against *Enterococcus fecalis*, and 1.56 μg·mL^−1^ and 6.25 μg·mL^−1^ against *Salmonella enteritidis* and *Pseudomonas aeruginosa*, respectively. The extracts of the studied species proved to be an alternative source in the search for new antimicrobial agents for the treatment of diseases caused by bacteria.

Overall, phytochemicals found in agri-food wastes have a significant potential to be used as alternative antibiotics and food additives. Additionally, their valorisation can lead to the production of value-added products with beneficial properties.

### 3.1. Nitrogen Alkaloids 

Alkaloids are a type of organic nitrogen heterocyclic compound that have a wide range of chemical structures based on the rings in the molecule [94]. Nicotine, morphine, caffeine, and mescaline are some of the well-known alkaloids. Plants produce alkaloids as a defence mechanism against insects and herbivores. These compounds also have antibacterial properties against a range of microorganisms, such as *Mycobacterium fortuitum*, *Mycobacterium tuberculosis*, *Mycobacterium smegmatis*, *E. coli*, *S. aureus*, *Salmonella typhimurium*, *Klebsiella pneumonia*, and *P. aeruginosa* [94,96].

The coffee industry generates a significant amount of by-products that can be used as a source of bioactive compounds [102,103]. Researchers have evaluated the antibacterial activity of arabica coffee leaves and found that the extracts contain the alkaloids trigonelline and caffeine [102]. These extracts were found to be effective against *E. coli*. 

### 3.2. Phenolic Compounds

Plant polyphenols, also known as phenolic compounds, are organic compounds that contain at least one phenol group and have an aromatic ring with one or more hydroxyl groups in their molecular structure [95], and they are classified into flavonoids and non-flavonoids based on their structural characteristics [94,96] secondary metabolites that play a crucial role in plant physiology, including defence against herbivores and pathogens and mechanical support for the plant. [94] have shown antimicrobial properties against a wide range of microorganisms, and they can sensitize multidrug-resistant strains to bacteriostatic or bactericidal antibiotics, making them promising natural antimicrobial agents [96]. Additionally, polyphenols have been established as chemopreventive and therapeutic agents due to their potential health-benefiting properties, including antioxidant, antiallergic, anti-inflammatory, anticancer, antihypertensive, and antimicrobial features [94,95,96]. Sharma et al. [108] investigated the biological activities of polyphenols in skinned fresh and ageing onions. The authors found that the antibiofilm activity against *E. coli*, *P. aeruginosa*, *S. aureus*, and *Bacillus cereus* increased with ageing onions as the levels of quercetin and total phenolic content also increased upon aging in the studied varieties.

#### 3.2.1. Flavonoids 

Plant flavonoids, which have a 2-phenyl-benzo-γ-pyrane nucleus with two benzene rings, have demonstrated promising antimicrobial activities and antioxidant properties [94,96]. Many classes of flavonoids, including flavonols, flavanols, flavanones, isoflavonoids, chalcones, and dihydrochalcones, have been identified as allelochemicals that inhibit microbial growth. Flavonoids are also known to inhibit quorum sensing and biofilm formation, as well as act as resistant-reversal agents [96]. Catechins and proanthocyanidins possess antioxidant properties and have been proposed to neutralize bacterial toxic factors originating from *V. cholerae*, *V. vulnificus*, *S. aureus*, *Bacillus anthracis*, and *C. botulinum*. Additionally, citrus flavonoids, such as apigenin, kaempferol, quercetin, and naringenin, are effective antagonists of cell–cell signalling [95,120]. Chrysin and kaempferol restrict the DNA gyrase activity, which is an essential enzyme in DNA replication in *E. coli*, while aglycone flavonoids, such as myricetin, hesperetin, and phloretin, inhibit biofilm formation in *Staphylococcus* strains [96]. 

#### 3.2.2. Non-Flavonoids

Phenolic acids, including benzoic, phenylacetic, and phenylpropionic acids, have been discovered to have inhibitory effects on both pathogenic and non-pathogenic bacteria and fungi. These include *E. coli*, *Lactobacillus* spp., *S. aureus*, *P. aeruginosa*, and *Candida albicans* [95,96]. 

Hydroxycinnamic acids, such as caffeic, coumaric, ferulic, and sinapic acids, have also been found to inhibit the growth of *Bacillus cereus*, *S. aureus*, and *Pseudomonas fluorescens* [95]. Ferulic acid and gallic acid have also demonstrated antibacterial properties against various bacterial isolates. Both acids damage the cell walls of *E. coli*, *P. aeruginosa*, and *S. aureus*, leading to local damage and cellular material leakage [96]; gallic acid has been shown to exhibit strong antibacterial potential against *Enterococcus faecalis*, *Streptococcus pneumonia*, *P. aeruginosa*, *Moraxella catarrhalis*, *S. aureus*, *Enterococcus faecalis*, *E. coli*, and *Streptococcus agalactiae* strains [96]. 

### 3.3. Terpenoids

Terpenoids are a diverse group of organic compounds that are similar to terpenes. They consist of mono- and sesquiterpenoids [94], which are the main components of essential oils. Essential oils are volatile plant products [96] that can be extracted from various plant parts, such as flowers and fruits. They contain a mixture of low-mass plant natural products or phytochemicals, including myrcene, *o*-cimene, citral, geraniol, eugenol, carvacrol, linalool, citronellal, carvone, limonene, terpinenes, menthol, and menthone [94,96].

Essential oils have strong antimicrobial properties and are commonly used in traditional medicine. They are considered safe for consumption and vital host tissues. However, their stability is crucial for their quality and pharmacological potency [96]. Essential oils are known for their remarkable antibacterial activities against both Gram-positive and negative pathogens, including bactericidal and re-potentiating or re-sensitizing of antibiotics potentials against pathogenic microbes. They have also demonstrated their potential in targeting and disturbing the most prevalent drug-resistance-determining mechanisms of microbes, namely the cell wall, cell membrane and permeability, drug efflux pumps, mobile genetic elements, quorum sensing, and biofilm [96].

Citrus fruits are the main source of essential oils [94,113,114,116]. For example, Djenane [113] evaluated the chemical composition of citrus peel (orange, lemon, and bergamot) essential oils. The essential oils analysed were mainly composed of limonene (77.4%) for orange essential oil; linalyl acetate (37.3%) and linalool (23.4%) for bergamot essential oil; and limonene (51.4%), β-pinene (17.0%), and γ-terpinene (13.5%) for lemon essential oil. The in vitro antimicrobial activity of the essential oils was evaluated against *S. aureus*, which revealed that lemon essential oil had more antibacterial effects than the other essential oils. 

### 3.4. Organosulfur Compounds 

Organosulfur compounds, also known as thiols, are present in various plants and vegetables. These compounds include glucosinolates and allyl sulphides, which contain sulfur in their structure. Glucosinolates are found in cruciferous vegetables of the *Brassicales* order while allyl sulphides are abundant in garlic [94]. 

Glucosinolates play a vital role in plant defence against microbial pathogens and insect herbivores. They act as signalling molecules that initiate pathways such as stomatal closure, apoptosis, and callose accumulation [121]. A study by Blažević et al. [118] investigated the glucosinolate profile and antibacterial activity of *Lepidium latifolium* L. against food spoilage bacteria. The results showed that allyl isothiocyanate, a compound found in the plant, was highly effective against *E. coli*. 

## 4. Potential Applications, Limitations, and Challenges for Antibacterial Phytochemicals from Agri-Food Wastes

The examples that are given in Table 2 point to the potential use of several antibacterial phytochemicals as an alternative to conventional antibiotics to treat antibiotic-resistant pathogen-originated infections, as well as an alternative to chemical additives to foodborne bacteria. These phytochemicals have demonstrated potential as bactericidal agents and have proved to inhibit the vital events for the sustenance and resistance of the pathogen, including efflux pumps, replication machinery, and cell permeability, among others. Additionally, their valorisation can lead to the production of other value-added products with beneficial properties spanning other fields of applications, such as cosmetics. Nevertheless, despite all the potential shown in the extraction of compounds from agri-food wastes to unveil new antibacterial compounds, there are several limitations and challenges to overcome. Agri-food wastes are complex mixtures containing compounds in a wide range of concentrations, and often, the bioactive compounds are present in very low amounts. Therefore, efficient extraction, purification, and characterization methods are required to obtain the active compounds from the waste materials. Additionally, the variability of the composition of agri-food wastes affects the quality and quantity of the extracted compounds. Furthermore, in many cases, the bioactive effect is not elicited by a single phytochemical but instead results from the synergistic effect of several compounds present in a single extract. This poses important constraints to the definition of sustainable and scalable production methods to ensure the availability of the extracted compounds for global health applications. For the new molecules identified with antibacterial activity, potential toxicity effects must be considered, which may limit their use in human and animal health applications. This requires further research and assays to determine the safety and efficacy of the extracted compounds. Furthermore, the lack of regulatory frameworks for the use of the new antibacterial compounds in human and animal health applications will delay their use for several years or decades. Finally, we have to consider the potential for the development of resistance to the extracted compounds, which may limit their long-term effectiveness against bacterial infections and result in the development of more aggressive forms of AMR.

## 5. Conclusions

Agri-food wastes uncover a plethora of naturally occurring phytochemicals that could hold significant bioactive potential for many uses in animal and human applications. Bacteria and other microbes are very relevant to human activity, including our metabolism. For this reason, growing AMR against antibiotics constitutes a severe health problem. To unveil new phytochemicals and methods able to mitigate this challenge, many researchers around the world have turned their attention towards delving into the composition of agri-food wastes. The exploration of this field can pave the way for novel and effective drugs against resistant bacteria and help to alleviate the AMR pressure in healthcare systems worldwide. This strategy is continuously driving the isolation and characterization in agri-food wastes of many promising antibacterial compounds from different chemical families, mainly nitrogen alkaloids, phenolic and organosulfur compounds, and terpenoids. Hopefully, some of these molecules will be effective against AMR.

## Figures and Tables

**Table 1:** Most important bacteria causing food poisoning, environmental hazards, or human health complications.

Bacteria	Reported Effects	Refs.
Food poisoning		
*Bacillus* *cereus*	Soil resident; pathogenic spectrum ranges from probiotic to lethal and highly toxic strains causing food poisoning (diarrhoea, nausea, and vomiting), but also local to severe systemic infections; forms spores; found in poorly processed foods, such as dairy products, cooked rice and pasta, meat, poultry, vegetables, and fruits	[11,30,31]
*Brucella* spp.	Gram-negative, non-spore-forming and nonencapsulated cocco bacilli; can infect animals and humans, causing wide clinical manifestations: intermittent fever, headache, nausea, vomiting, night sweats, progressive deterioration in visual function, periorbital pain, and other impacts in hepatobiliary, genitourinary, musculoskeletal, cardiovascular, and integumentary systems; transmitted to humans mainly through contaminated raw milk and dairy products	[12,32,33]
*Campylobacter* spp.	Commensal bacteria (microaerophilic Gram-negative, non-spore-forming), often present in the intestine of many animals (cattle, sheep, poultry, pets, and wild animals); common cause of food poisoning, causing diarrhoea, cramping, and fever; found in raw or undercooked poultry, unpasteurized milk, and contaminated water	[13,34,35]
*C. difficile*	Anaerobic toxigenic bacterium ubiquitous in the environment; can colonise the intestinal tract of animals and humans, causing severe infectious colitis (severe colon diarrhoea and inflammation leading to significant morbidity and mortality worldwide); detected in different meats, fish, fruits, and vegetables	[14,36,37]
*C. botulinum*	Genetically and ecologically diverse strains; produce a potent neurotoxin that can cause botulism (muscle weakness, paralysis, and even death); detected in improperly stored food and feed raw materials	[38,39,40]
*E. coli*	Some strains produce toxins (e.g., Shiga toxins) that can cause bloody diarrhoea, kidney failure, and infections in the urinary tract, bloodstream, and central nervous system; reported in ground beef, unpasteurized milk, and fresh produce	[15,41,42]
*Listeria* *monocytogenes*	Can cause severe infections in animals and humans, particularly in pregnant women, newborns, and people with weakened immune systems; symptoms are very diverse and may include fever, muscle and headaches, neck stiffness, abdominal cramps, diarrhoea, nausea, vomiting, septicaemia, and even meningitis; found in deli meats, hot dogs, soft cheeses, and smoked seafood	[21,22,43]
*Salmonella* spp.	Can affect food humans and animals, leading to diarrhoea, fever, and abdominal cramps; *S. enterica* species can cause other symptoms, such as enteric fever, enterocolitis with diarrhoea, bacteraemia (bacterial infection in the blood), and chronic asymptomatic carriage; often found it in poultry, eggs, and raw meat, but also in contaminated produce such as sprouts or melons	[19,20,44]
*S. aureus*	These bacteria can produce a toxin that can cause food poisoning when food is left at room temperature for too long; its effects on human health range from minor skin infections to severe tissue infection and sepsis; mainly found in meat and poultry and dairy products	[16,45,46]
*Vibrio* spp.	Found in saltwater, causing infections by ingestion of contaminated seafood; *V. parahaemolyticus* and *V. vulnificus* are the leading causes of seafood-associated infections and mortality in the United States; symptoms include diarrhoea, vomiting, fever, wound infections, and septicaemia; detected in raw or undercooked seafood, particularly oysters	[23,24,47]
*V. cholerae*	The toxigenic strains of serogroups O1 and O139 can cause cholera, a severe diarrheal disease that can be life-threatening if left untreated; detected in infected shells of crabs, shrimps, and other shellfish	[25,48,49]
*Yersinia* spp.	Yersiniosis, particularly from *Y. enterocolitica*, causes diarrhoea and abdominal pain; detected in raw or poorly processed foods (sashimi from fish and cattle liver, raw tako-octopus, semi boiled pig’s ear), contaminated raw milk, food, and feed raw materials contamination by rodents	[17,18,50]
Human pathogens		
*Acinetobacter* *baumannii*	Nosocomial pathogen responsible for most hospital-acquired nosocomial infections (ventilator-associated, as well as bloodstream infections) in critically ill patients	[26,51,52]
*Klebsiella* *pneumoniae*	Gram-negative opportunistic pathogen, infecting critically ill and immunocompromised patients and causing different infectious diseases, including urinary tract infections, bacteraemia, pneumonia, and liver abscesses	[27,53,54]
*Mycobacterium tuberculosis*	Can cause tuberculosis, a serious infectious disease that primarily affects the lungs, being the leading cause of death due to a single infection agent	[29,55,56]
*Neisseria* *meningitidis*	An exclusively human pathogen that can cause meningitis, a serious infection of the membranes that surround the brain and spinal cord	[57,58,59]
*Neisseria* *gonorrhoeae*	The host-adapted human pathogen causing gonorrhoea (a sexually transmitted infection that may lead to pelvic inflammatory disease and infertility)	[60,61,62]
*P. aeruginosa*	Opportunistic bacteria that can cause recurrent infections in humans (pneumonia, urinary tract infections and bacteraemia), particularly in people with cystic fibrosis and weakened immune systems; it can also cause food spoilage and it is resistant to many antibiotics; found in water, soil, and human hosts	[28,63,64]
*Streptococcus* *pyogenes*	Gram-positive bacteria that can cause several diseases such as strep throat, acute pharyngitis, scarlet fever, or skin and soft-tissue infections, especially necrotizing fasciitis	[65,66,67]

**Table 2:** Antibacterial potential of phytochemicals found in agri-food wastes.

Phytochemicals	Agri-Food Waste	Target Pathogen	Action	Ref.
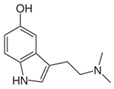 Nitrogen alkaloids				
Imidazole	*Pilocarpus pennatifolius* (jaborandi) leaves	Gram-positive (*Enterococcus fecalis)* and Gram-negative (*Salmonella enteritidis*, *P. aeruginosa*)	Antibacterial	[101]
Trigonelline, caffeine	Arabica coffee leaves	Gram-positive (*S. aureus*) and Gram-negative (*E. coli*, *P. aeruginosa*)	Antibacterial and bactericidal	[102]
Caffeine, quinine	Coffee silverskin extracts	Gram-positive (*S. aureus*) and Gram-negative (*E. coli*, *P. aeruginosa*)	Antibiofilm	[103]
Peganine, harmol, harmine, β-carboline, quinazoline alkaloids	*Peganum harmala* seeds	*Ralstonia solanacearum* Phylotype II, *Pectobacterium cartovorum* subsp. *Cartovorum, Erwinia amylovora, Burkholderia gladioli* pv. *allicola*	Antibacterial	[104]
Solanidine, α-chaconine, α-solanine	Potato peels	*Lactobacillus reuteri*, *Lactobacillus acidophilus*, *Lactobacillus rhamnosus*, *E. coli*	Antimicrobial	[105]
α-Solanine, α-chaconine	Potato sprouts	Gram-positive bacteria (*S. aureus*, *Bacillus subtilis*, *Enterococcus hirae*) and Gram-negative (*E. coli*, *P. aeruginosa*)	Antimicrobial	[106]
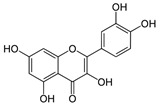 Phenolic compounds				
Ellagic acid, trans-fertaric acid, quercetin, kaempferol	Grape pomace and lees	Gram-positive (*S. aureus*, *Bacillus subtilis*, *Bacillus cereus*) and Gram-negative (*E. coli*)	Bactericidal	[107]
Quercetin and its glucosides (quercetin aglycone, quercetin-4′-*O*-monoglucoside, quercetin-3,4′-*O*-diglucoside, anthocyanin)	Skinned onions	Gram-positive (*S. aureus*, *Bacillus cereus*) and Gram-negative (*E. coli*, *P. aeruginosa*)	Antibacterial and antibiofilm	[108]
Sinensetin, 4′,5,6,7-tetramethoxyflavone, nobiletin, tangeretin, 3,3′,4′,5,6,7-hexamethoxyflavone, 3,3′,4′,5,6,7,8-heptamethoxyflavone, eriocitrin, nairutin, hesperidin	Orange peels	*E. coli*, *S. aureus*, *Bacillus subtilis*	Antibacterial	[109]
Hesperidin, eriocitrin, diosmin	Lemon peels	*S. aureus*	Antibacterial	[110]
Pelargonidin-diglucoside, gallotannin, ellagitannin, cyanidin-glucoside, pelargonidin-glucoside, catechin, p-coumaroyl-glucoside, *p*-coumaroyl-ester, *p*-coumaroyl-glucoside, quercetin-rutinoside, ellagic acid, quercetin-glucoside, quercetin-glucuronide, methyl-ellagic acid-pentose, and kaempferol-glucuronide.	*Camellia oleifera* seeds	*E. coli*, *S. aureus*, *Bacillus subtilis*	Antibacterial	[111]
Chlorogenic acid, caffeic acid, coumaric acid	Apple, lime, grapes, pomegranate, and papaya wastes	*Bacillus subtilis*, *E. coli*	Antibacterial activity of pigments	[112]
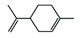 Terpenoids				
Limonene, linalyl acetate, linalool, β-pinene, γ-terpinene	Citrus peels (orange, lemon, and bergamot)	*S. aureus*	Bactericidal	[113]
Limonene, β-myrcene, linalool, α-pinene, β-pinene	Orange peels	Gram-positive (*S. aureus*, *Listeria monocytogenes*) and Gram-negative (*E. coli*, *P. aeruginosa*)	Bactericidal	[114]
Caffeic acid, *p*-coumaric acid, salicylic acid	Yellow passion fruit pulp and seeds	Gram-positive (*S. aureus*, *Bacillus cereus*) and Gram-negative (*E. coli*, *Salmonella enteritidis*)	Antibacterial	[115]
D-limonene, lauric acid, 1-methyl-1,4-cyclohexadiene, methyl linoleate, myristic acid, (E,E,E)-2,6,10-trimethyl-2,6,9,11-dodecanetetraen-1-al, palmitic acid, β-myrcene	Orange peels	*Cutibacterium acnes* (formerly *Propionibacterium acnes*)	Antibacterial	[116]
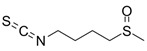 Organosulfur compounds				
Glucoraphanin, sulforaphane, sulforaphane nitrile	*Punica granatum* L. peel	Gram-positive (*S. aureus*, *Enterococcus faecalis*) and Gram-negative (*P. aeruginosa*, *Klebsiella pneumoniae*)	Antibacterial	[117]
Allyl isothiocyanate	*Lepidium latifolium* flower, leaf, stem, and root	Gram-positive (*Listeria monocytogenes*, *S. aureus*) and Gram-negative (*Salmonella* Typhimurium, *E. coli*, *P. aeruginosa*)	Antibacterial a (time-killing and growth kinetic assays)	[118]
Lucoraphanin, sulforaphane, sulforaphane nitrile	Broccoli (raw, cooked, and cooked broccoli plus mustard seeds as a source of myrosinase)	*E. coli*	Antibacterial	[119]
